# CTCF binding site sequence differences are associated with unique regulatory and functional trends during embryonic stem cell differentiation

**DOI:** 10.1093/nar/gku470

**Published:** 2014-06-02

**Authors:** Robert N. Plasschaert, Sébastien Vigneau, Italo Tempera, Ravi Gupta, Jasna Maksimoska, Logan Everett, Ramana Davuluri, Ronen Marmorstein, Paul M. Lieberman, David Schultz, Sridhar Hannenhalli, Marisa S. Bartolomei

The eighth author's name was spelled incorrectly upon publication of this paper. The correct name is Ronen Marmorstein.

